# Advances in the Development of Mitochondrial Pyruvate Carrier Inhibitors for Therapeutic Applications

**DOI:** 10.3390/biom15020223

**Published:** 2025-02-03

**Authors:** Henry Politte, Lingaiah Maram, Bahaa Elgendy

**Affiliations:** 1 Department of Anesthesiology, Washington University School of Medicine, St. Louis, MO 63110, USA; henry.politte@slu.edu (H.P.); lmaram@wustl.edu (L.M.); 2 Center for Clinical Pharmacology, Washington University School of Medicine and University of Health Sciences and Pharmacy, St. Louis, MO 63110, USA

**Keywords:** MPC (mitochondrial pyruvate carrier), pyruvate transport, energy metabolism, metabolic disorders (e.g., diabetes, obesity, and cancer), neurodegenerative disorders, therapeutic target, MPC inhibitors, drug design, virtual screening, medicinal chemistry

## Abstract

The mitochondrial pyruvate carrier (MPC) is a transmembrane protein complex critical for cellular energy metabolism, enabling the transport of pyruvate from the cytosol into the mitochondria, where it fuels the citric acid cycle. By regulating this essential entry point of carbon into mitochondrial metabolism, MPC is pivotal for maintaining cellular energy balance and metabolic flexibility. Dysregulation of MPC activity has been implicated in several metabolic disorders, including type 2 diabetes, obesity, and cancer, underscoring its potential as a therapeutic target. This review provides an overview of the MPC complex, examining its structural components, regulatory mechanisms, and biological functions. We explore the current understanding of transcriptional, translational, and post-translational modifications that modulate MPC function and highlight the clinical relevance of MPC dysfunction in metabolic and neurodegenerative diseases. Progress in the development of MPC-targeting therapeutics is discussed, with a focus on challenges in designing selective and potent inhibitors. Emphasis is placed on modern approaches for identifying novel inhibitors, particularly virtual screening and computational strategies. This review establishes a foundation for further research into the medicinal chemistry of MPC inhibitors, promoting advances in structure-based drug design to develop therapeutics for metabolic and neurodegenerative diseases.

## 1. Introduction

The mitochondrial pyruvate carrier (MPC) is a hetero-dimeric protein complex located within the inner mitochondrial membrane (IMM), which plays a crucial role in cellular metabolism. This membrane-bound complex is responsible for the transport of pyruvate, the final product of glycolysis, across the IMM and into the mitochondrial matrix [1]. In this section, we will dive deeper into the structure, composition, and localization of MPC, providing a solid foundation for understanding its biological function and relevance in metabolic and neurodegenerative disorders.

### 1.1. Structure and Composition

The MPC complex comprises two main subunits, MPC1 and MPC2, which assemble into a functional unit responsible for transporting pyruvate into the mitochondria [2]. Both MPC1 and MPC2 are integral membrane proteins with multiple transmembrane domains, enabling them to span the IMM and facilitate pyruvate translocation. These subunits exhibit a high degree of sequence conservation across various species, underscoring their essential role in cellular metabolism [3].

Recent mutagenesis experiments and homology modeling have highlighted the importance of specific amino acid residues in MPC function. Notably, the Phe66 residue in MPC1, as well as Asn100 and Lys49 in MPC2, have been identified as critical to molecular binding [4]. These residues are thought to contribute to the formation of a substrate-binding cavity or to directly interact with the pyruvate substrate. The hydrophobic nature of the Phe66 residue in MPC1 likely supports the creation of an environment conducive to pyruvate binding and transport. In MPC2, the polar Asn100 may contribute to a hydrogen bonding network within the substrate binding site, while the positively charged Lys49 could interact with the carboxylate group of pyruvate, stabilizing the binding interaction. These residues are thought to contribute to the formation of a substrate-binding cavity or to directly interact with the pyruvate substrate. The hydrophobic nature of Phe66 in MPC1 likely supports the creation of an environment conducive to pyruvate binding and transport.

In addition to the canonical MPC1 and MPC2 subunits, recent studies have identified additional MPC orthologs, such as MPC3 and MPC4, which display distinct tissue-specific expression patterns. These orthologs may modulate the activity of the MPC complex in a context-dependent manner, potentially influencing pyruvate transport in specific physiological or pathological states [5,6,7]. Further research is needed to elucidate the functional implications of these orthologs and to investigate their roles in the regulation of MPC-mediated pyruvate transport.

### 1.2. Localization

**Figure 1:**
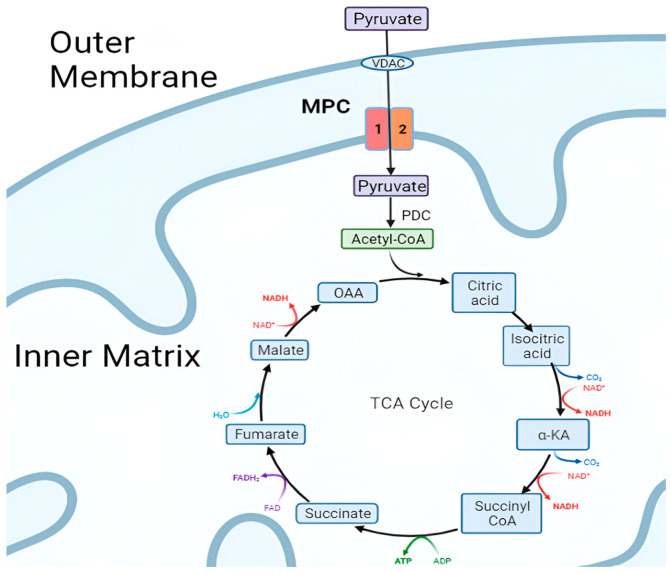
Overview of MPC localization and function. MPC facilitates the transport of pyruvate into the inner matrix. Pyruvate is then converted to acetyl-CoA by the pyruvate dehydrogenase complex (PDC). Citrate (CIT), isocitrate (ICIT), α-ketoglutarate (α-KA) (also known as 2-oxoglutarate), succinyl-CoA (SUC-CoA), succinate (SUC), fumarate (FUM), malate (MAL), and oxaloacetate (OAA).

MPC is embedded within the IMM (Figure 1), which separates the mitochondrial matrix from the intermembrane space [8]. This specific localization is essential for the proper functioning of the complex, as it facilitates the efficient transport of pyruvate into the mitochondrial matrix, where pyruvate acts as a crucial substrate for several metabolic pathways. The IMM is highly impermeable to ions and metabolites, requiring specialized transporters like the MPC to facilitate the regulated transport of specific molecules across the membrane [9].

The targeting of MPC subunits to the IMM is directed by conserved amino acid sequences within their transmembrane domains, which function as mitochondrial targeting signals [10]. Once imported into the mitochondria, the MPC1 and MPC2 subunits assemble into a functional complex, embedded within the IMM. This complex is strategically positioned near other key components of mitochondrial metabolism, including the pyruvate dehydrogenase complex and enzymes of the tricarboxylic acid cycle, facilitating efficient metabolic integration.

The structure, composition, and precise localization of the MPC complex underscore its essential role in cellular metabolism and highlight its potential as a therapeutic target for treating metabolic disorders. Gaining a detailed understanding of the MPC complex and its interactions with other mitochondrial components is crucial for the rational design and development of novel MPC inhibitors with enhanced efficacy and selectivity. For a more comprehensive review of MPC’s structure, composition, and biological function, readers are encouraged to refer to the recent work by Tuvolari et al. [11], which provides in-depth insights into the MPC complex.

## 2. Biological Functions of MPC

**Figure 2:**
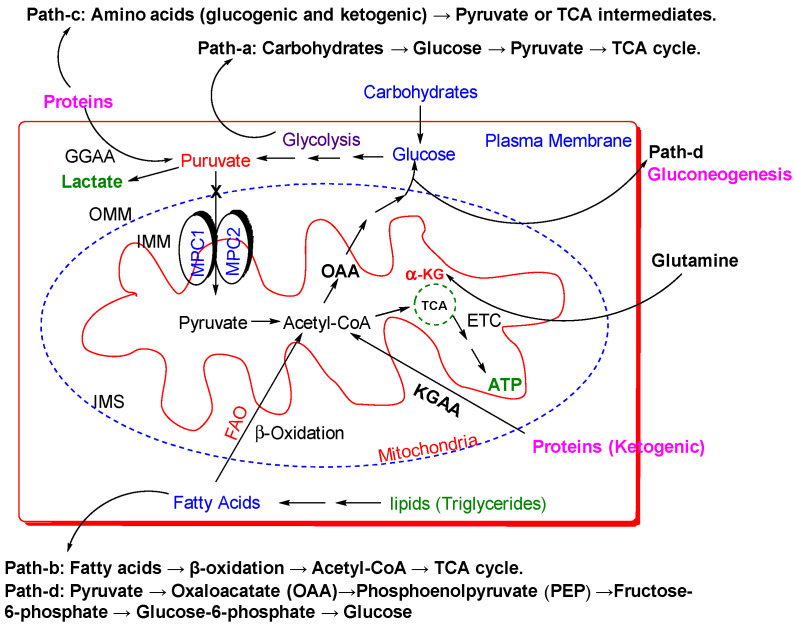
Diagram illustrating the structure of mitochondria with MPC proteins located in the IMM, the metabolic pathway of pyruvate in mitochondria, and other related metabolic processes such as fatty acid synthesis, protein metabolism, and gluconeogenesis.

The primary function of the MPC is to facilitate the transport of pyruvate into the mitochondrial matrix, where it serves as a crucial substrate for multiple metabolic pathways (Figure 2). By mediating pyruvate entry across the IMM, MPC plays an essential role in regulating mitochondrial respiration, metabolic flux, and overall cellular metabolism. In this section, we will explore the biological functions of MPC, highlighting its central role in coordinating and integrating metabolic processes.

### 2.1. Pyruvate Transport and Mitochondrial Respiration

Pyruvate, the end product of glycolysis, is an essential substrate for mitochondrial oxidative metabolism. Initially, pyruvate is transported through the outer mitochondrial membrane into the inter-membrane space by a voltage-dependent anion channel (VDAC). From there, the MPC facilitates its transport across the IMM into the mitochondrial matrix. Once inside the matrix, pyruvate undergoes oxidative decarboxylation by the pyruvate dehydrogenase complex (PDC), yielding acetyl-CoA, which enters the tricarboxylic acid (TCA) cycle [12,13]. The TCA cycle, in turn, generates reducing equivalents in the form of NADH and FADH2, which drive the electron transport chain, ultimately producing adenosine triphosphate (ATP), the primary energy currency of the cell.

By controlling pyruvate availability for oxidative metabolism, MPC acts as a key gatekeeper for mitochondrial respiration and cellular energy production. Inhibition or downregulation of MPC activity can reduce pyruvate import into the matrix, potentially limiting ATP production and impacting cellular energetics.

### 2.2. Metabolic Flux Regulation

Beyond the central role in regulating mitochondrial respiration, MPC contributes to maintaining metabolic homeostasis by modulating the balance between different metabolic pathways, including glycolysis, fatty acid oxidation, and gluconeogenesis (Figure 2) [14]. By controlling pyruvate entry into the mitochondrial matrix, MPC indirectly influences the activity of these pathways, playing a pivotal role in the overall regulation of cellular metabolism.

A reduction in MPC activity and the resulting decrease in pyruvate availability for mitochondrial oxidation can trigger a compensatory increase in fatty acid oxidation [15]. This shift may enhance the production of ketone bodies and alter lipid metabolism, with potential implications for metabolic health.

MPC’s control over the pyruvate influx into the mitochondria also impacts glycolysis and gluconeogenesis [16]. By modulating mitochondrial pyruvate levels, MPC indirectly affects the cytosolic pyruvate pool, thereby influencing the glycolytic rate. In conditions such as fasting or intense exercise, when gluconeogenesis is upregulated, the availability of mitochondrial pyruvate, partly regulated by MPC, can impact glucose production [17].

As pyruvate is a primary input for the TCA cycle, the regulation of its transport into the mitochondrial matrix by MPC has direct implications on the rate of this vital metabolic pathway [18]. The TCA cycle, in turn, provides energy-rich molecules, such as ATP, and intermediates for biosynthetic pathways. By controlling pyruvate transport, MPC can thus influence cellular energy production and biosynthesis [19].

Pyruvate also serves as a substrate in transamination reactions, contributing to the metabolism of several amino acids [20]. By regulating mitochondrial pyruvate levels, MPC influences the availability of pyruvate for these reactions, impacting amino acid metabolism.

Interestingly, while pyruvate is not directly involved in fatty acid oxidation, MPC activity indirectly affects this process [21]. High pyruvate levels, facilitated by MPC, promote its conversion to acetyl-CoA, which enters the TCA cycle, thereby reducing the need for acetyl-CoA derived from fatty acid oxidation.

Post-translational modifications (PTMs) are dynamic, covalent alterations to proteins that regulate their activity and stability [22,23]. Lysine acetylation of MPC subunits significantly reduces pyruvate transport efficiency. In diabetic heart systems, acetylation of MPC2 at lysine 19 and 26 reduces pyruvate transport efficiency by 70% [24]. In neurons, acetylation of MPC1 at lysine 45 and 46 impairs mitochondrial pyruvate uptake, and acetyl-mimetic mutants confirm the functional significance of this modification in regulating synaptic transmission [25]. Sirtuin 3 (SIRT3) counteracts this modification by deacetylating MPC [26]. This deacetylation protects against cardiac hypertrophy and ischemia–reperfusion injury [27]. PTMs also affect MPC subunit stability, with acetylation leading to decreased subunit abundance [28]. Additionally, glucose rerouting due to altered MPC activity can elevate O-GlcNAcylation, contributing to maladaptive cellular growth, particularly in heart failure and other pathologies [25].

## 3. The Role of MPC in Metabolic Disorders, Neurodegenerative Diseases, and Clinical Significance

Dysregulation of MPC function has been implicated in the pathogenesis of various metabolic disorders, including type 2 diabetes, obesity, and non-alcoholic fatty liver disease (NAFLD) [29], as well as in heart failure [24]. In these conditions, changes in MPC expression or activity led to impaired pyruvate transport and disruptions in mitochondrial metabolism. Emerging evidence highlights the critical role of MPC in neurodegenerative diseases, demonstrating its importance in maintaining neuronal energy homeostasis and mitochondrial function, which are key factors in understanding conditions such as Alzheimer’s disease and Parkinson’s disease. In this section, we will discuss the pathophysiological implications of MPC dysregulation in metabolic disorders and neurodegeneration, as well as its clinical significance in the context of therapeutic interventions.

### 3.1. MPC in Type 2 Diabetes

Type 2 diabetes is characterized by insulin resistance and impaired glucose homeostasis, resulting in chronic hyperglycemia. Emerging evidence suggests that alterations in MPC activity may contribute to the development of insulin resistance and the progression of type 2 diabetes [30]. In vivo studies have shown that treatment with the MPC inhibitor MSDC-0602 improves insulin sensitivity and glycemic control in humans [31]. A Phase 2b clinical trial involving patients with NASH reported that MSDC-0602K, the potassium salt form of MSDC-0602, led to statistically significant reductions in hemoglobin A1c (HbA1c) levels, fasting plasma glucose, and fasting plasma insulin, indicating enhanced insulin sensitivity and improved glycemic control.

Reduced MPC expression or activity has been observed in diabetic animal models and human subjects, resulting in impaired mitochondrial pyruvate transport and a metabolic shift toward increased glycolysis, gluconeogenesis, and fatty acid oxidation [32]. Dysregulation of MPC in type 2 diabetes may exacerbate insulin resistance by limiting pyruvate availability for mitochondrial oxidation and promoting the accumulation of metabolic intermediates, such as diacylglycerols and ceramides, which are known to interfere with insulin signaling pathways [33]. Furthermore, reduced MPC activity may enhance hepatic glucose production via increased gluconeogenesis, further exacerbating hyperglycemia. These findings underscore the potential of targeting MPC as a therapeutic strategy in type 2 diabetes to restore metabolic balance and improve insulin sensitivity.

### 3.2. MPC in Obesity

Obesity, a major risk factor for type 2 diabetes and other metabolic disorders, is characterized by an excessive accumulation of adipose tissue and disrupted metabolism. Emerging evidence suggests that MPC expression and activity are modulated by nutritional status and adiposity, with reduced MPC function observed in models of high-fat diet-induced obesity. In a recent in vivo study, MPC inhibitors such as Zaprinast and 7ACC2 were shown to improve glucose tolerance in diet-induced obese mice, highlighting their potential as therapeutic agents [34].

The downregulation of MPC in obesity may contribute to insulin resistance by promoting a metabolic shift toward increased fatty acid oxidation and the accumulation of lipid intermediates that interfere with insulin signaling pathways. Furthermore, impaired MPC function may exacerbate adipose tissue dysfunction and inflammation, two key factors in the development of metabolic complications associated with obesity. These findings underscore the potential of targeting MPC to mitigate metabolic dysfunction and improve outcomes in obesity-related disorders.

### 3.3. MPC in Non-Alcoholic Fatty Liver Disease

Non-alcoholic fatty liver disease (NAFLD) is characterized by the excessive accumulation of lipids in the liver, which can progress to more severe conditions such as non-alcoholic steatohepatitis (NASH), fibrosis, cirrhosis, and even hepatocellular carcinoma [35]. Dysregulation of MPC has been implicated in the pathogenesis and progression of NAFLD, with reduced MPC expression and activity observed in both animal models and human cases [36]. In vivo studies have demonstrated that MPC inhibitors, such as MSDC-0602 and DRX-065, may alleviate disease symptoms, underscoring their potential as therapeutic agents for the treatment of NAFLD [37].

Impaired MPC function in NAFLD may exacerbate hepatic steatosis by limiting pyruvate availability for mitochondrial oxidation, thus redirecting metabolic flux toward lipogenesis and triglyceride synthesis [38]. Moreover, reduced MPC activity may contribute to mitochondrial dysfunction, oxidative stress, and inflammation, which are pivotal drivers of NAFLD progression and associated liver injury. These findings highlight the importance of MPC in maintaining hepatic metabolic balance and suggest a promising therapeutic strategy for mitigating NAFLD and its complications.

### 3.4. MPC in Neurodegenerative Diseases

**Figure 3:**
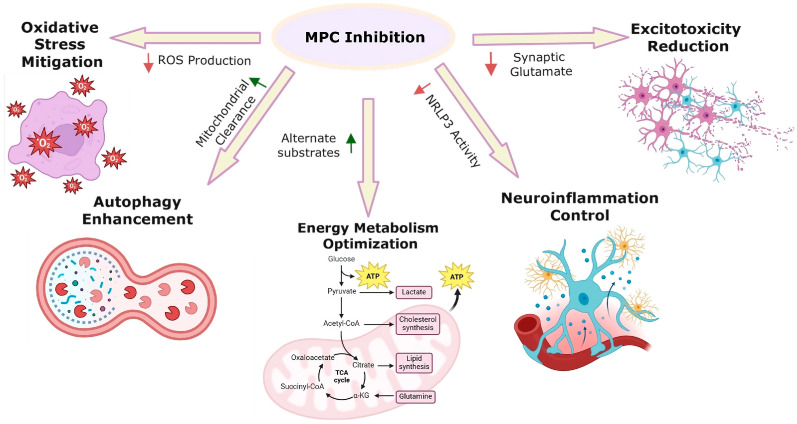
Potential pathways of MPC inhibition in neurodegenerative disorders.

Neurodegenerative diseases, including Alzheimer’s disease (AD), Parkinson’s disease (PD), Huntington’s disease (HD), and amyotrophic lateral sclerosis (ALS), are characterized by progressive neuronal loss and inflammation often linked to disruptions in mitochondrial metabolism [39,40]. The pivotal role of MPC in regulating metabolic pathways has sparked interest in its therapeutic potential, particularly its viability as a drug target without compromising ATP production or TCA cycle functionality (Figure 3).

Studies have revealed surprising metabolic flexibility in cells with inhibited MPC activity, where alternative pathways can adapt to sustain TCA cycle function and support cellular growth [41]. For example, MPC inhibition activates glutamate dehydrogenase (GDH), redirecting glutamine metabolism to maintain the TCA cycle and lipid synthesis [42].

Recent evidence suggests that MPC inhibition could offer therapeutic benefits for neurodegenerative diseases [43,44,45,46,47,48,49]. In AD, the accumulation of amyloid-beta and Tau proteins is a hallmark of disease pathology. A study by Ceyzeriat and coworkers demonstrated that knocking out or inhibiting MPC in astrocytes reduced amyloid-beta and Tau aggregates in the brain [50]. This reduction in neurofibrillary tangles and amyloid deposits points to a potential therapeutic approach for slowing the progression of AD pathology.

Similarly, research by Ghosh et al. showed that the insulin sensitizer MSDC-0160, a thiazolidinedione (TZD) class MPC inhibitor, protected against neuronal degradation in mouse and rat models exposed to neurotoxic compounds [51]. The neuroprotective effects of MSDC-0160 are thought to involve modulation of the mammalian target of rapamycin (mTOR) pathway, which enhances autophagy—a process linked to benefits in aging and neurodegeneration [52,53]. It was found that knocking out various components of the mTOR pathway (AKT-1, RHEB-1, or LET-363) in nematodes disrupted the neuroprotective effects of MSDC-1060 on dopaminergic neurons expressing the A53T α-synuclein [54]. This highlights that MPC modulation downregulates the mTOR pathway. Therefore, MPC inhibition can stimulate autophagy.

Additionally, Divakaruni et al. explored the impact of MPC inhibition on excitotoxic neuronal death in rat cortical neurons. They found that treatment with the MPC inhibitor UK-5099 maintained neuronal viability for over three days, even with reduced pyruvate oxidation [55]. The neurons adapted by oxidizing non-glucose substrates, such as leucine and β-hydroxybutyrate, which sustained TCA cycle activity and mitigated glycolytic and ATP production changes. Interestingly, in nutrient-rich conditions, glucose-derived carbon incorporation into the TCA cycle decreased without significant lactate accumulation or increased oxidation of alternative substrates, highlighting metabolic flexibility in response to MPC inhibition.

Cells increase reliance on alternative fuels, such as fatty acids and amino acids, to maintain energy production when pyruvate transport is compromised [19,56,57]. This shift can help maintain mitochondrial function but also induces oxidative stress by promoting excess ROS production, particularly through altered TCA cycle activity [58].

In conditions like Parkinson’s disease, oxidative stress plays a role in neuronal damage. MPC inhibition can enhance oxidative stress by increasing the reliance on pathways like glutaminolysis and fatty acid oxidation. These processes increase mitochondrial ROS production, which, although adaptive at first, can lead to cellular damage if not tightly regulated [57,59]. The increased ROS levels, though contributing to neuronal stress, may also activate protective responses, such as the unfolded protein response, which helps manage the oxidative burden [57].

The dysregulation of mitochondrial function due to MPC inhibition can affect autophagy pathways. In neurodegenerative diseases, defective mitophagy (a selective form of autophagy) exacerbates mitochondrial dysfunction. For example, in Parkinson’s disease, mutations in genes like parkin, which play a role in mitophagy, can worsen the impact of oxidative stress on neurons [60].

Although these findings underscore the potential of MPC as a therapeutic target for neurodegenerative diseases, further research is needed to elucidate its precise mechanisms of action in disease models. Additionally, these therapeutic approaches must be validated through human clinical trials to evaluate their safety and efficacy.

### 3.5. Clinical Significance and Therapeutic Potential

The pivotal role of MPC dysregulation in metabolic disorders has positioned it as a promising therapeutic approach. Pharmacological modulation of MPC activity, whether through enhancement or selective inhibition, offers potential to ameliorate the metabolic imbalances characteristic of type 2 diabetes, obesity, and NAFLD.

In preclinical models, MPC inhibitors have demonstrated substantial therapeutic benefits. These include improvements in insulin sensitivity, reductions in hepatic steatosis, and attenuation of inflammation, highlighting their therapeutic potential for metabolic and neurodegenerative disorders. Specifically, in type 2 diabetes models, MPC inhibition has been shown to lower blood glucose levels, improve glucose tolerance, and enhance insulin sensitivity [61]. Similarly, in NAFLD models, MPC inhibitors have reduced hepatic lipid accumulation and inflammation, providing evidence of their utility in ameliorating disease progression [62].

Studies show that high glucose levels increase the acetylation of MPC2, impairing its normal function [63]. This disruption causes pyruvate accumulation in the cytoplasm, decreases mitochondrial membrane potential, and leads to mitochondrial damage, ultimately inducing apoptosis. Additionally, inhibition or knockdown of MPC2 has been shown to exacerbate mitochondrial dysfunction and apoptotic pathways in podocytes, indicating the role of MPC in maintaining mitochondrial health and preventing apoptosis under metabolically stressful conditions [63].

Many cancer cells rely heavily on aerobic glycolysis to meet their energy and biosynthetic demands [64]. This metabolic reprogramming often involves reduced mitochondrial pyruvate transport via MPC downregulation, allowing cancer cells to avoid oxidative stress and apoptosis while enhancing lactate production to support the tumor microenvironment.

Beyond its pharmacological significance, MPC expression and activity have been proposed as potential biomarkers for diagnosis and monitoring of metabolic conditions. Changes in MPC expression or activity in response to treatment could serve as indicators of therapeutic efficacy or disease progression, offering a valuable tool for personalized medicine [65]. Moreover, deepening our understanding of the molecular mechanisms regulating MPC and its role in metabolic disorders could unveil new druggable targets within affected pathways. This knowledge could pave the way for the development of more precise and effective treatment strategies tailored to the specific metabolic or neurodegenerative conditions of individual patients.

## 4. Medicinal Chemistry Approaches to MPC Inhibition

The therapeutic potential of the mitochondrial pyruvate carrier (MPC) in treating metabolic disorders and neurodegenerative diseases underscores the pressing need for the development of potent, selective, and clinically relevant MPC inhibitors. Achieving this goal demands a multidisciplinary approach within medicinal chemistry, leveraging structure-based drug design and structure–activity relationship (SAR) studies. These efforts must be guided by detailed insights into the mechanisms and characteristics of known MPC inhibitors. This section delves into these critical aspects, aiming to advance the development of effective MPC-targeting therapeutics and unlock new possibilities for treating complex metabolic and neurodegenerative conditions.

### 4.1. Structure-Based Drug Design and Ligand-Based Virtual Screening

Structure-based drug design leverages detailed molecular and structural information about a biological target to guide the development of novel therapeutics [66]. For MPC, the lack of a resolved crystal structure poses a significant challenge. However, advances in homology modeling and mutagenesis studies have provided critical insights into the structural features of MPC [67]. With the advent of advanced machine learning algorithms, such as AlphaFold, newly predicted MPC structures may provide valuable models for virtual screening and the identification of potential inhibitors [68].

In cases where structural information is limited, ligand-based virtual screening provides an effective alternative route for identifying novel MPC inhibitors [69]. This approach relies on the structural characteristics of known active compounds rather than the target’s structure, making it particularly suited to the current understanding of MPC. Among ligand-based virtual screening methods, developing pharmacophore hypothesis models is widely employed [70]. A pharmacophore represents the spatial and electronic features required for a molecule to optimally interact with a specific biological target, facilitating activation or inhibition. Key features of pharmacophores typically include hydrogen bond donors or acceptors, aromatic rings, and other functional groups involved in critical interactions with the target site.

Additionally, computational techniques like molecular docking and molecular dynamics simulations can enhance predictions of potential binding sites and interactions between MPC and small-molecule inhibitors [71]. These methods are invaluable for characterizing inhibitor–target interactions, though their accuracy remains constrained by the absence of a crystal structure for MPC. When integrated with ligand-based virtual screening, these computational approaches provide a robust framework for identifying and designing novel MPC inhibitors, advancing therapeutic development for metabolic and neurodegenerative disorders.

### 4.2. SAR and QSAR Studies

The development of MPC inhibitors relies heavily on understanding the intricate relationship between chemical structure and biological activity. Structure–activity relationship (SAR) studies involve the systematic modifications of lead compounds to assess how changes in their chemical structure influence biological activity. These studies help identify critical structural features essential for MPC inhibition, providing valuable insights into the molecular mechanisms of MPC inhibition and informing the design of inhibitors with improved pharmacological profiles [72].

Complementing traditional SAR approaches, quantitative structure–activity relationship (QSAR) analyses offer a more in-depth understanding of structural optimization [73]. QSAR models establish quantitative relationships between the physicochemical properties or theoretical molecular descriptors of compounds and their biological activities. These models allow for the prediction of activity in novel compounds and guide candidate selection and refinement. Classical QSAR methods such as linear regression, multiple linear regression (MLR), and partial least squares (PLS) provide a foundation for analysis, while advanced machine learning techniques, such as support vector machines (SVM) and artificial neural networks (ANN), significantly enhance predictive accuracy [74,75]. Moreover, the three-dimensional QSAR (3D-QSAR) model, which incorporates steric effects, electrostatic interactions, and hydrophobicity, delivers more precise predictions and deeper insights into the molecular determinants of activity [76,77]. In the absence of a crystal structure for MPC, ligand-based QSAR models are particularly useful, aiding in the identification and optimization of new inhibitors.

Together, SAR and QSAR studies, when combined with robust biological assays, serve as essential tools for identifying and optimizing MPC inhibitors. These approaches help identify off-target effects and potential liabilities, ultimately enhancing the therapeutic index of MPC inhibitors. By providing a systematic and quantitative framework, SAR and QSAR analyses advance the rational design and selection of promising drug candidates, driving progress in MPC-targeting therapeutics.

### 4.3. Known MPC Inhibitors

A common approach in medicinal chemistry is to leverage insights from the structures and target interactions of known inhibitors to guide the design of new therapeutics. This approach is particularly valuable for ligand-based virtual screening, which relies on detailed knowledge of active compound structures [4]. Several MPC inhibitors have been reported in the literature, including UK5099, Rosiglitazone, GW604714X, GW450863X, Lonidamine, and Zaprinast [78,79,80]. A thorough understanding of these inhibitors’ chemical structures, mechanisms of action, and pharmacological properties can guide the design and development of next-generation MPC inhibitors with enhanced therapeutic profiles. An exploration of the structural development and classification of MPC-known inhibitors will be covered in Section 5.

In developing MPC-targeting therapeutics, these medicinal chemistry approaches are critical. A robust understanding of structure-based drug design, SAR studies, and known MPC inhibitors will enable the refinement of MPC inhibitors, ultimately aiding in the treatment of metabolic and neurodegenerative diseases. Future research in this field holds promise for creating novel MPC-targeting therapeutics with the potential to transform the clinical management of these conditions.

## 5. Recent Advances in the Medicinal Chemistry of MPC Inhibitors

Given the central role of the MPC in metabolic regulation and its involvement in various metabolic disorders, MPC has emerged as an attractive target for the development of novel therapeutics. In recent years, several small-molecule MPC inhibitors have been identified, with promising therapeutic effects demonstrated in cellular and animal models [79,81]. In this section, we will discuss the progress made in the development of MPC-targeting therapeutics, the challenges faced, and future directions for research in this area.

### 5.1. Small-Molecule MPC Inhibitors: Discovery and Development

**Figure 4:**
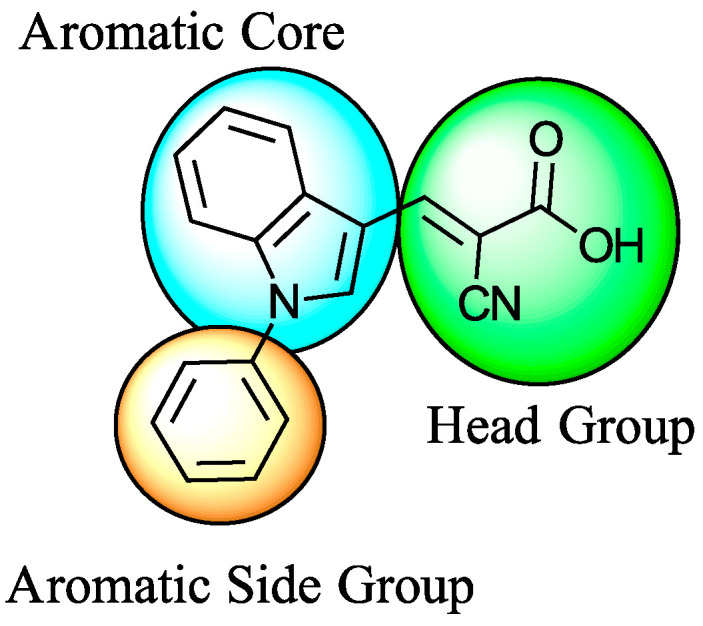
Structure of UK-5099. Cyanoacetic acid head group (green). Indole aromatic core (blue). Phenyl aromatic side group (orange).

Several small-molecule inhibitors of MPC, such as UK-5099 (Figure 4) [82] and MSDC-0602 [83], have been identified, offering insights into the structure–activity relationships and pharmacological properties of these compounds. These inhibitors effectively modulate MPC activity, resulting in a metabolic shift favoring glycolysis, fatty acid oxidation, and gluconeogenesis. In preclinical models of type 2 diabetes and NAFLD, MPC inhibitors have shown promise in enhancing insulin sensitivity, reducing hepatic steatosis, and attenuating inflammatory responses.

Structural similarities among known MPC inhibitors suggest a classification based on shared features, offering a framework for systematic exploration and design. To fully realize their therapeutic potential, it is crucial to investigate the diverse structural classes of MPC inhibitors. Each class is defined by unique characteristics that influence inhibitor efficacy, selectivity, and pharmacological profiles. Such exploration will not only deepen our understanding of MPC inhibition mechanisms but also guide the development of more effective and targeted therapeutics.

### 5.2. UK-5099 (Cyano-Cinnamate) Derivatives

UK-5099 is widely regarded as the gold standard in MPC inhibition, serving as a foundational compound for research into the therapeutic potential of targeting the mitochondrial pyruvate carrier. Known for its potent inhibitory activity, the structure of UK-5099 has inspired a range of derivatives, each designed to enhance the original compound’s efficacy or address its limitations.

Derivatives of UK-5099 are structurally composed of three key regions (Figure 4). The α-cyano-cinnamate head group, an aromatic core, and an aromatic side group. The head group, resembling pyruvate, is critical for MPC recognition of the inhibitor. While ester derivatives of this moiety, such as BE1988 and JXL011, do not inhibit MPC in vitro, they may offer advantages as prodrugs for in vivo drug delivery.

The aromatic core is one of the most varied regions across this class of MPC inhibitors, encompassing both bicyclic structures (e.g., indoles, azaindoles, etc.) and monocyclic cores (e.g., benzenes, furans, pyrazoles, thiophenes). This core can also be modified with additional substituents; however, the removal of the aromatic core significantly diminished potency. This is exemplified by α-cyano-4-methyl-2-pentanoate (Entry 6, Table 1), which was found to be inactive.

By blocking pyruvate transport into mitochondria, UK-5099 inhibits mitochondrial oxidative phosphorylation and enhances glycolysis [84]. Halestrap’s 1974 study on structural modifications of UK-5099 highlighted the essential role of the nitrile group and the aromatic core in MPC inhibition, as shown by high IC_50_ values in analogs like α-cyano-4-methyl-2-pentanoate, α-fluorocinnamate, and α-thio-2-furanpyruvate [85]. Halestrap proposed that the hydrophobic aromatic moiety stabilizes the cyanoacetic acid moiety in a favorable binding orientation within the MPC.

Recent computational studies by Hegazy et al. [4] and Tavoulari et al. [86] have independently identified a new scaffold of MPC inhibitors. This scaffold retains the cyano-cinnamate moiety but expands the structure to include a furan and an additional aromatic ring. Initial screenings indicate that these novel compounds exhibit nanomolar IC_50_ values, suggesting promising inhibitory potency.

Our group recently developed a novel MPC inhibitor scaffold incorporating a pyrazole ring into the aromatic core. This series demonstrated high potency against MPC, combined with excellent solubility and metabolic stability. Notably, these compounds showed no affinity for PPAR-γ and were active in attenuating hepatic stellate cell activation, suggesting potential in hepatic disease management [87]. SAR studies indicated a preference for hydrophobic groups in the aromatic side chain, with substituents such as dibromo-phenyl, dichloro-phenyl, trifluoromethylphenyl, and naphthyl groups being well tolerated.

Another series of UK-5099 derivatives was reported by Jung and coworkers for the treatment of hair loss [88]. Known as the JXL series (entries 9–18, Table 1), these inhibitors aim to restrict pyruvate entry into mitochondria, thereby diverting pyruvate towards alternative pathways, such as lactate production via lactate dehydrogenase (LDH). Studies demonstrated a correlation between LDH activity and hair follicle stem cell activation, suggesting that MPC inhibition may support hair regrowth through this mechanism. These compounds retain the indole core of UK-5099 while extending the aromatic side group by one carbon. They found that a 3,5-di(trifluoromethyl)benzene side group significantly improved potency in MPC oxygen consumption assays. Additional modifications to the head group, including diethyl (cyanomethyl) phosphates, thiazolidinediones, and bis-nitriles, were explored but did not enhance MPC inhibition.

In traditional medicinal chemistry, α-cyano-cinnamate derivatives have typically been deemed unsuitable for further development, due to the cyanoacetic–acetic acid moiety’s double bond functioning as a Michael acceptor. This characteristic raises concerns about off-target interactions, prompting research into reversible cysteine-targeting approaches. A study by Serafimova et al. showed that electron-deficient olefins, including acrylamides, could be chemically modified to reversibly interact with cysteine thiols [89].

Interestingly, introducing a nitrile group enhanced the reactivity of these olefins while simultaneously preventing the formation of irreversible adducts. Interestingly, the molecular structures analyzed in this study were notably similar to those investigated by Halestrap in 1979. Building on these insights, our group recently conducted a similar NMR study using a pyrazole-based scaffold, observing comparable reversibility [90]. These findings indicate a low propensity for Michael acceptor binding, suggesting minimal covalent interactions with off-target proteins. This approach provides a promising avenue for designing MPC inhibitors with a reduced risk of unintended interactions.

**Table 1:** α-Cyano-cinnamate MPC inhibitors.

Entry	Name	Structure	IC_50_	Bioassay	Ref.
1.	UK-5099	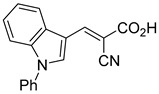	0.140 μM	Oxygen consumption rate	[88]
2.	α-Cyano-cinnamate	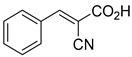	200.0 nM	Oxygen uptake	[9]
3.	α-Cyano-4-hydroxycinnamate	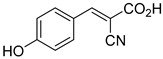	1.5 μM	Oxygen uptake	[9]
4.	α-Cyano-3-hydroxycinnamate	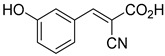	1.5 μM	Oxygen uptake	[9]
5.	α-Cyano-5-phenyl-2,4-pentadienoate	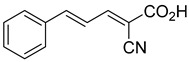	200 nM	Oxygen uptake	[9]
6.	α-Cyano-4-methyl-2-pentanoate	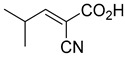	i.a.	Oxygen uptake	[9]
7.	α-Fluorocinnamate	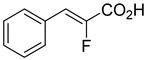	i.a.	Oxygen uptake	[9]
8.	α-Thio-2-furanpyruvate	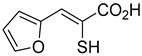	i.a.	Oxygen uptake	[9]
9.	JXL011	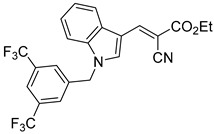	Not Reported	N/A	[88]
10.	JXL020	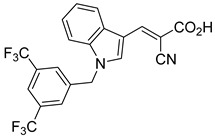	16.6 nM	Oxygen consumption rate	[88]
11.	JXL069	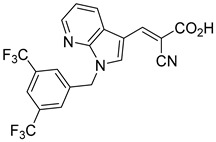	42.8 nM	Oxygen consumption rate	[88]
12.	JXL050	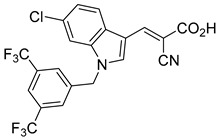	Not Reported	N/A	[88]
13.	JXL051	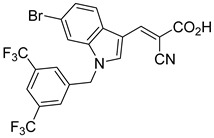	Not Reported	N/A	[88]
14.	JXL052	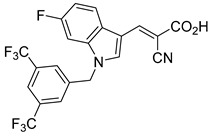	Not Reported	N/A	[88]
15.	JXL086	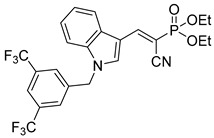	Not Reported	N/A	[88]
16.	JXL094	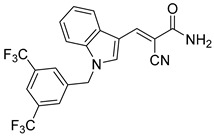	Not Reported	N/A	[88]
17.	JXL095	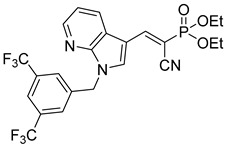	Not Reported	N/A	[88]
18.	BE1976	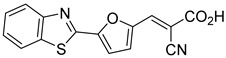	33.0 nM	Pyruvate OCR	[4]
19.	BE1978	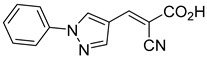	117 nM	Pyruvate OCR	[4]
20.	BE1980	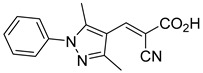	162 nM	Pyruvate OCR	[4]
21.	BE1984	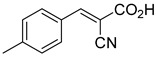	1.533 μM	Pyruvate OCR	[4]
22.	BE1985	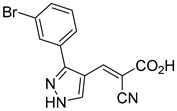	638 nM	Pyruvate OCR	[4]
23.	BE1988	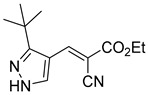	i.a.	Pyruvate OCR	[4]
24.	BE2617	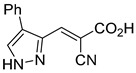	39 nM	Pyruvate OCR	[4]
25.	BE2623	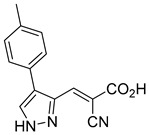	731 nM	Pyruvate OCR	[4]
26.	BE1975	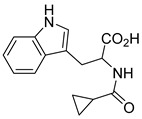	i.a.	Pyruvate OCR	[4]
27.	Compound 2	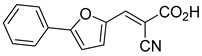	12.4 nM	Pyruvate transport inhibition	[86]
28.	Compound 3	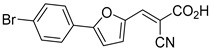	Not Reported	N/A	[86]
29.	Compound 4	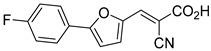	Not Reported	N/A	[86]
30.	Compound 5	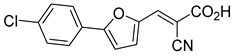	Not Reported	N/A	[86]
31.	Compound 6	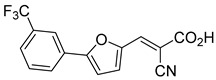	Not Reported	N/A	[86]
32.	Compound 7	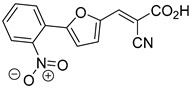	5.4 nM	Pyruvate transport inhibition	[86]
33.	Compound 8	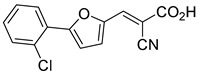	Not Reported	N/A	[86]
34.	Compound 9	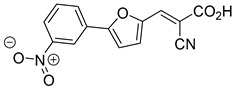	Not Reported	N/A	[86]
35.	BE2625	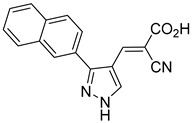	212.0 nM	Pyruvate OCR	[87]
36.	BE2639	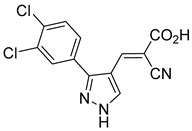	107.0 nM	Pyruvate OCR	[87]
37.	BE2645	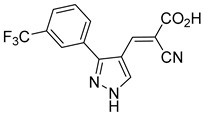	108.0 nM	Pyruvate OCR	[87]
38.	BE2647	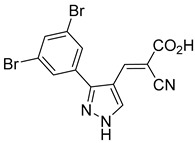	70.0 nM	Pyruvate OCR	[87]
39.	BE2648	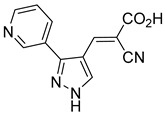	7.771 μM	Pyruvate OCR	[87]
40.	BE2650	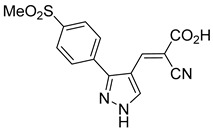	18.26 μM	Pyruvate OCR	[87]
41.	BE2659	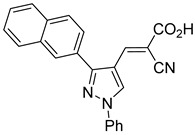	789 nM	Pyruvate OCR	[87]

### 5.3. Thiazolidinediones

Thiazolidinediones (TZDs) are a class of drugs historically recognized for their role as insulin sensitizers in the treatment of type 2 diabetes [90,91]. Their primary mechanism of action involves functioning as agonists for peroxisome proliferator-activated receptor-gamma (PPAR-γ). However, PPAR-γ activation has been linked to several adverse side effects, including hypertension, bone loss, and heart failure, prompting the withdrawal of TZDs from the market in several countries. Interestingly, some TZDs have also been found to exhibit an inhibitory effect on MPC, offering potential for alternative therapeutic applications. For example, Rosiglitazone, a TZD previously approved by the FDA, was found to induce a rapid dose-dependent reduction in pyruvate-driven, uncoupler-simulated respiration in C2C12 myoblasts [81]. A similar effect was observed with UK-5099, further supporting the notion that TZDs can effectively inhibit MPC.

Among TZD derivatives, MSDC-0160 (Mitoglitazone), developed by Metabolic Solutions Development Company, was reported to modulate both PPAR-γ and MPC activity. A related compound, MSDC-0602K, exhibited reduced affinity for PPAR-γ while maintaining comparable binding affinity for MPC. This profile positions MSDC-0602K as a promising candidate with the potential to minimize PPAR-γ-related side effects while retaining effective MPC modulation [92].

**Figure 5:**
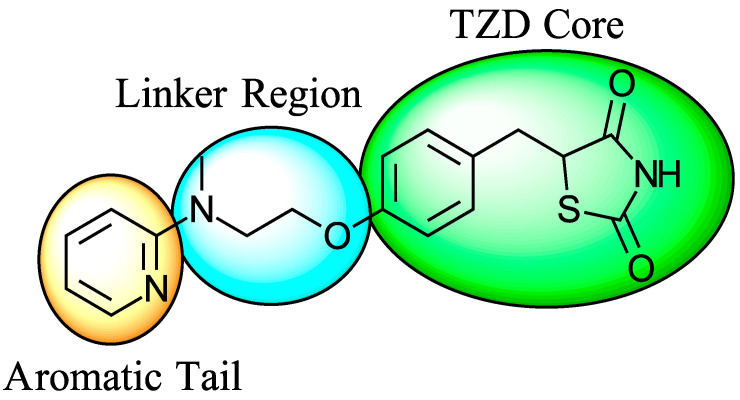
Structure of Rosiglitazone. Thiazolidinedione core (green). Linker region (blue). Aromatic tail (orange).

Central to every TZD molecule is the thiazolidinedione ring, a five-membered ring structure containing one sulfur atom and two oxygen atoms, which defines the TZD class (Figure 5). Typically, TZDs feature one or more aromatic rings attached to the core thiazolidinedione structure extending from the 5th position on the ring. This aromatic group is commonly a phenyl ring, often modified with various substituents.

Additionally, many TZDs include a linker region that connects the core ring to a secondary aromatic system. This linker generally consists of ether and/or ketone chains, typically spanning from four to five carbons in length. The “tail” region of TZDs is usually composed of substituted aromatic rings, such as benzene or pyridine. The 5th position of the thiazolidinedione ring also functions as a stereocenter. While most TZDs are administered as a racemic mixture, recent work has explored methods to control or eliminate stereochemistry, potentially enhancing their pharmacological properties [93].

Compounds such as (*E*)-5-(4-hydroxybenzylidene) thiazolidine-2,4-dione (Entry 1, Table 2) and (*E*)-5-(3-hydroxy-4-mthoxybenzylidene) thiazolidine-2,4-dione (Entry 2, Table 2) were reported to have similar inhibitory effects on mitochondrial respiration similar to UK-5099. Additionally, a deuterated (*R*)-Pioglitazone, which utilizes deuterium to establish the stereochemistry of the fifth position, has shown markedly reduced binding affinity for PPAR-γ and is currently under clinical evaluations, albeit not as an MPC inhibitor [94].

**Table 2:** Thiazolidinedione (TZD) MPC inhibitors.

Entry	Name	Structure	IC_50_	Bioassay	Ref.
1.	(*E*)-5-(4-Hydroxybenzylidene) thiazolidine-2,4-dione	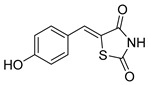	762.0 nM	Mitochondrial Respiration	[93]
2.	(*E*)-5-(3-Hydroxy-4-Methoxy benzylidene) thiazolidine-2,4-dione	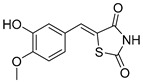	Not reported	N/A	[93]
3.	PXL065	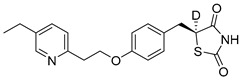	Not reported	N/A	[94]
4.	Rosiglitazone	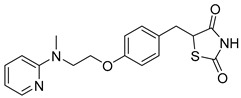	1.18 μM	Binding affinity to mouse MPC	[81]
5.	MSDC-0602K	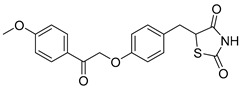	N/A	N/A	[92]
6.	Pioglitazone	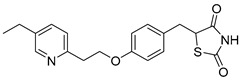	1.20 μM	Binding affinity to mouse MPC	[81]
7.	Mitoglitazone (MSDC-0160)	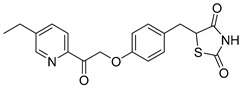	1.20 μM	Displacement of photoprobe from MPC	[81]
8.	MSDC-1437	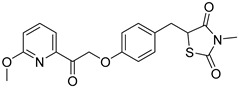	i.a.	……	[92]
9.	GW450863X	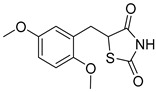	Not reported	N/A	[95]
10.	GW504714X	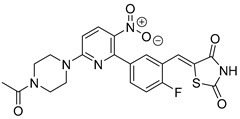	Not reported	N/A	[95]
11.	Nitrofurantoin	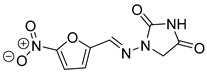	3.3 μM	Pyruvate transport inhibition	[86]

### 5.4. Miscellaneous MPC Inhibitors

Over the years, several other structural classes have been identified as active MPC inhibitors (Table 3). Lonidamine (Entry 1, Table 3), an anti-cancer drug, is known to enhance tumor sensitivity to chemotherapy, hyperthermia, and radiotherapy. Its primary mechanism of action includes the inhibition of MPC, monocarboxylate transporter (MCT), and succinate–ubiquinone reductase activities. Studies have confirmed that Lonidamine effectively inhibits MPC activity in isolated rat liver mitochondria [95].

Zaprinast, (Entry 2, Table 3) originally developed as a phosphodiesterase 5/6 (PDE5/PDE6) inhibitor and the precursor to sildenafil (Viagra), has also been identified as an MPC inhibitor [96]. Zaprinast inhibits pyruvate-driven oxygen consumption in brain mitochondria and disrupts MPC function in liver mitochondria. However, there has been limited exploration of Zaprinast derivatives specifically for MPC inhibition. Finck et al. further confirmed Zaprinast’s ability to inhibit MPC activity and showed it can improve glucose tolerance in diet-induced obese mice. Zaprinast’s effects extend beyond hepatic MPC inhibition, indicating peripheral metabolic benefits potentially linked to other mechanisms, such as increased glucose uptake in skeletal muscle or its action as a phosphodiesterase (PDE) inhibitor. These findings underscore Zaprinast’s multifaceted metabolic influence, albeit with potential off-target effects that may limit its direct therapeutic use [34].

Compound 7ACC2 (Entry 4, Table 3) is recognized for its potent monocarboxylate transporter (MCT) inhibition [97]. Its primary function involves blocking extracellular lactate uptake into the mitochondria of cancer cells, ultimately inducing cell death. Moreover, 7ACC2 has been identified as a potent MPC inhibitor, effectively suppressing hepatic gluconeogenesis and improving glucose tolerance in diet-induced obese mice. Unlike Zaprinast, the effects of 7ACC2 are specifically tied to hepatic MPC2 activity, with minimal peripheral action observed. This specificity makes 7ACC2 a promising tool for investigating MPC inhibition and its role in metabolic regulation. However, limitations such as poor solubility and a short half-life pose significant challenges for its therapeutic application.

Recently, Schumacher and coworkers developed a series of novel cancer therapeutics derived from 7ACC2 [98]. Among these, compounds FACC2 (Entry 6, Table 3) and FACC3 (Entry 7, Table 3) demonstrated sub-micromolar potency in pyruvate-driven OCR assays, while FACC1 (Entry 5, Table 3) showed activity with an IC_50_ above 1 μM. These derivatives feature 4-fluorophenyl expansions on the amino moiety of 7ACC2, enhancing their activity.

Using a bioluminescence-based MPC activity assay, Finck et al. screened a chemical library and identified 35 novel potential MPC modulators [42]. Employing a pharmacophore model based on 7ACC2, they prioritized hits for further investigation. Among these, 7ACC1, carsalam, and six quinolone antibiotics (Entries 3, 8–14, Table 3) were found to share a structural pharmacophore with 7ACC2. Experimental validation confirmed these compounds as MPC inhibitors. Specifically, mitochondrial respiration studies using pyruvate as a substrate demonstrated that nalidixic acid, 7ACC1, and carsalam were the most effective inhibitors at a concentration of 10 μM. Dose–response analyses revealed that 7ACC1 was nearly as potent as 7ACC2, with an IC50 comparable to UK-5099, while nalidixic acid and carsalam exhibited approximately 50% inhibition at the same concentration. Further, 7ACC1, nalidixic acid, and carsalam potently suppressed glucose production in isolated hepatocytes at 10 μM concentrations. Notably, nalidixic acid improved glucose tolerance in vivo in obese mice. These findings highlight the therapeutic potential of targeting MPC for diabetes treatment and provide scaffolds for developing potent, novel MPC inhibitors.

Kunji et al. identified entacapone (Entry 16, Table 3), a drug commonly used in Parkinson’s disease therapy, as a novel MPC inhibitor [86]. Using a refined pharmacophore model, entacapone was shown to fit the essential binding motifs of MPC inhibitors. Thermostability assays demonstrated significant shifts in the melting temperature of the MPC complex, indicating strong binding affinity. Functional validation through a [^14^C]-pyruvate homo-exchange assay with MPC1L/MPC2 proteoliposomes confirmed its inhibitory activity, with an IC_50_ of approximately 630 nM. These findings underscore entacapone’s potential off-target effects and suggest its utility as a scaffold for developing new MPC inhibitors.

Recent work from Wenes et al. introduced MITO-66 (Entry 17, Table 3) as a novel MPC inhibitor designed to enhance the efficacy of CD19-CAR T cells in antitumor treatment [99]. MITO-66 inhibits MPC with an IC_50_ of 119 nM, though its molecular structure has not been reported. The compound was shown to induce a stem cell-like memory phenotype in CAR T cells derived from healthy donors and patients with relapsed/refractory B-cell malignancies. CAR T cells conditioned with MITO-66 demonstrated superior antitumor activity, effectively controlling human pre-B cell acute lymphoblastic leukemia in mouse models. These cells retained a memory phenotype after transfer, provided protection against tumor rechallenge, and outperformed clinical-stage AKT and PI3Kδ inhibitors in a leukemia stress model. This study provides compelling preclinical evidence supporting the use of MITO-66 during CAR T cell manufacturing to significantly enhance antitumor efficacy.

**Table 3:** Miscellaneous MPC inhibitors.

Entry	Name	Structure	IC_50_	Bioassay	Ref.
1.	Lonidamine	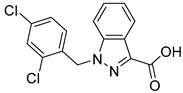	4.6 μM	Pyruvate transport inhibition	[86,95]
2.	Zaprinast	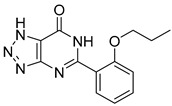	321 nM	Pyruvate transport inhibition	[86,98]
3.	7ACC1	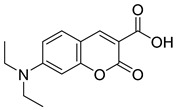	Not reported	Oxygen consumption rate	[34]
4.	7ACC2	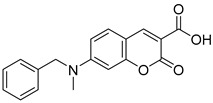	Not reported	Oxygen consumption rate	[34]
5.	FACC1	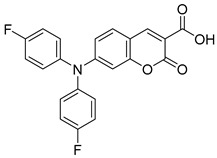	>1 μM	Pyruvate-driven oxygen consumption	[97]
6.	FACC2	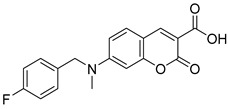	16.6 nM	Pyruvate-driven oxygen consumption	[97]
7.	FACC3	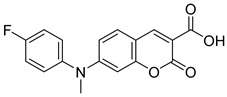	147.6 nM	Pyruvate-driven oxygen consumption	[97]
8.	Carsalam	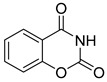	Not reported	RESPYR	[34]
9.	Clinafloxacin	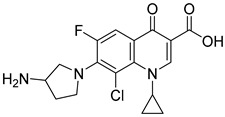	Not reported	RESPYR	[34]
10.	Sarafloxacin	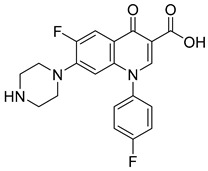	Not reported	RESPYR	[34]
11.	Nadifloxacin	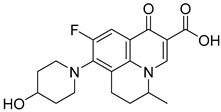	Not reported	RESPYR	[34]
12.	Pefloxacine	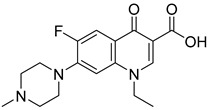	Not reported	RESPYR	[34]
13.	Nalidixic Acid	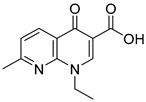	Not reported	RESPYR	[34]
14.	Moxifloxican	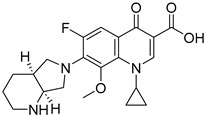	Not reported	RESPYR	[34]
15.	Entacapone	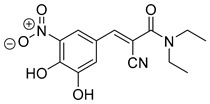	630.0 nM	Puruvate transport inhibition	[86]
16.	MITO-66	Not Reported	119 nM	Oxygen	[99]

## 6. Conclusions and Perspectives

The development of optimized MPC inhibitors represents a promising but challenging frontier in the treatment of metabolic and neurodegenerative diseases. Achieving high potency and selectivity remains critical to minimizing off-target effects and potential toxicities. A deeper exploration of the SAR of MPC inhibitors is essential to identify compounds with improved selectivity, efficacy, and safety profiles.

As discussed, TZDs can bind both MPC and PPAR-γ. While their potential as MPC inhibitors remains underexplored, their high affinity for PPAR-γ complicates further development within this class due to significant off-target effects. Future efforts should focus on optimizing TZD-based MPC inhibitors to enhance selectivity for MPC while reducing PPAR-γ binding, thus mitigating associated adverse effects.

The optimization of ADME properties of MPC inhibitors is another critical priority to ensure adequate drug exposure at target sites and to minimize systemic side effects. Furthermore, given MPC’s central role in cellular metabolism and energy homeostasis, the long-term safety and tolerability of MPC inhibitors must be rigorously evaluated in both preclinical and clinical settings.

To accelerate therapeutic advancements targeting MPC, the following key research directions should be pursued: (1) Structural Insights: Determining the crystal structure of the MPC complex would provide a deeper understanding of its molecular architecture, enabling rational drug design through structure-based approaches, as discussed earlier. (2) Innovative Screening: High-throughput screening and computational approaches can be employed to discover novel scaffolds beyond those covered in this review. (3) Medicinal Chemistry: Advances in medicinal chemistry are essential to refine current scaffolds and develop next-generation inhibitors with superior properties.

MPC-targeting therapeutics hold significant promise for treating various metabolic and neurodegenerative disorders, including type 2 diabetes, obesity, NAFLD, AD, PD, and ALS. By resolving current challenges in MPC inhibitor development and pursuing these innovative research avenues, the translation of basic research findings can be significantly accelerated, offering hope to patients afflicted with these debilitating conditions.

This review has provided a comprehensive overview of the mitochondrial pyruvate carrier (MPC), covering its structure, composition, localization, and key biological functions.

We have highlighted the clinical relevance of MPC dysregulation in metabolic and neurodegenerative disorders and underscored its potential as a therapeutic target. Finally, we reviewed recent advances in the development of MPC-targeting therapeutics, addressed existing challenges, and proposed future research priorities to guide ongoing and future studies in this exciting field. This section is not mandatory but can be added to the manuscript if the discussion is unusually long or complex.

## Data Availability

No new data were created or analyzed in this study.

## Abbreviations

The following abbreviations are used in this manuscript:

AD	Alzheimer’s disease
ADME	absorption, distribution, metabolism, and excretion
ALS	amyotrophic lateral sclerosis
ATP	adenosine triphosphate
HbA1c	hemoglobin A1c
HD	Huntington’s disease
PD	Parkinson’s disease
PDC	pyruvate dehydrogenase complex
PPAR-γ	peroxisome proliferator-activated receptor gamma
IFD	induced fit docking
OMM	outer mitochondrial membrane
IMS	inner mitochondrial space
IMM	inner mitochondrial matrix
α-KG	α-ketoglutarate
FAO	fatty acid oxidation
OAA	oxaloacetate
ETC	electron transport chain
GGAA	glucogenic amino acid
KGAA	ketogenic amino acid
MCT	monocarboxylate transporter
MPC	mitochondrial pyruvate carrier
mTOR	mechanistic target of rapamycin
NAFLD	non-alcoholic fatty liver disease
NASH	non-alcoholic steatohepatitis
QSAR	quantitative structure–activity relationship
SAR	structure–activity relationship
TZD	thiazolidinedione
VDAC	voltage-dependent anion channel
